# Supplementary material for: MetaRibo-Seq measures translation in microbiomes
Source: Nat Commun. 2020 Jun 29;11:3268. doi: 10.1038/s41467-020-17081-z (PMC7324362; doi:10.1038/s41467-020-17081-z)
Supplement: Supplementary file 10 — Supplementary Data 7 [file 41467_2020_17081_MOESM10_ESM.zip › File2/Confidence_High_Taxonomy/309570_out.krona.html]

Javascript must be enabled to view this page.

members
magnitude
magnitudeUnassigned
count
unassigned
taxon
rank

309570\_out

2

2
superkingdom
2

1239
2
phylum

2
class
186801

186802
order
2

186803
family
2

2
genus
2569097

2

SRS017521\_contig\_number\_47670SRS019068\_contig\_number\_contig-100\_685.607203
species
39488
